# Left-right asymmetric expression of the *Nodal-Lefty-Pitx2* module in developing turtle forebrain

**DOI:** 10.3389/fcell.2022.929808

**Published:** 2022-10-21

**Authors:** Eriko Kajikawa, Toru Miki, Masayoshi Takeda, Hiroshi Kiyonari, Hiroshi Hamada

**Affiliations:** ^1^ Laboratory for Organismal Patterning, RIKEN Center for Biosystems Dynamics Research, Hyogo, Japan; ^2^ Himeji City Aquarium, Himeji, Japan; ^3^ Laboratory for Animal Resources and Genetic Engineering, RIKEN Center for Biosystems Dynamics Research, Hyogo, Japan

**Keywords:** brain, left-right asymmetry, Nodal, reptile, turtle

## Abstract

The epithalamus of zebrafish shows morphological and molecular left-right (L-R) asymmetry, but such asymmetry is not apparent in tetrapods. To provide further insight into the evolutionary diversity of brain L-R asymmetry, we have now examined the developing brains of reptile embryos for expression of *Nodal*, *Lefty*, and *Pitx2*. Two turtle species, the Chinese softshell turtle and the red-eared slider turtle, showed left-sided expression of these three genes in the developing forebrain, with this expression occurring after *Nodal* expression at the lateral plate and the L-R organizer has disappeared. Nodal activity, as revealed by the detection of phosphorylated Smad2/3, was also apparent in the neural epithelium on the left side in both turtle species. In the Chinese softshell turtle, the habenula did not show apparent asymmetry in size and the parapineal organ was absent, but the expression of *Kctd12* in the habenula showed a small yet reproducible asymmetry. In contrast to the turtles, L-R asymmetric expression of *Nodal*, *Lefty*, *Pitx2*, or *Kctd12* was not detected in the developing brain of the Madagascar ground gecko. The transcriptional enhancer (ASE) responsible for the asymmetric expression of *Nodal*, *Lefty*, and *Pitx2* was conserved among reptiles, including the Chinese softshell turtle and Madagascar ground gecko. Our findings suggest that *Nodal*, *Lefty*, *and Pitx2* have the potential to be asymmetrically expressed in the developing brain of vertebrates, but that their expression varies even among reptiles.

## Introduction

Left-right (L-R) asymmetry of the brain is evident at anatomic and functional levels. However, the evolution of brain asymmetry and its functional importance remain largely unknown. Most of the progress in the characterization of such mechanisms has been made with the zebrafish epithalamus, a forebrain region that includes the centrally located pineal organ, the left-sided parapineal organ, and the bilateral habenular nuclei (Concha et al., 2000; Liang et al., 2000; Bianco and Wilson, 2009; Aizawa et al., 2011; Roberson and Halpern, 2018). *Nodal*, *Lefty*, and *Pitx2* genes are expressed simultaneously and asymmetrically on the left side of the developing diencephalon, with such expression determining the direction of epithalamus laterality (Roussigne et al., 2009; Signore et al., 2016). For example, whereas the parapineal is located on the left side of the pineal organ in wild-type zebrafish, the position of the parapineal organ is randomized in *Nodal* mutants (Concha et al., 2000; Liang et al., 2000). The *Nodal-Lefty-Pitx2* cassette is also expressed asymmetrically in the diencephalon of basal vertebrates such as lamprey and catshark (Lagadec et al., 2015).

The morphological and molecular asymmetry of the epithalamus varies among vertebrates (Concha and Wilson, 2001). Morphological asymmetry is apparent in cyclostomes (such as lamprey), chondrichthyans (such as catshark), and actinopterygians (such as zebrafish), but not in tetrapods including mammals. Asymmetric *Nodal* expression has been detected in teleosts, lamprey, and catshark but not in tetrapods, suggesting that it is an ancient feature of vertebrates but was lost during evolution. To clarify the evolutionary conservation of molecular brain asymmetry among vertebrates, we have now examined *Nodal*, *Lefty*, and *Pitx2* expression in the developing brain of reptiles, including two turtles and a gecko. Unexpectedly, we found that *Nodal*, *Lefty*, and *Pitx2* are expressed asymmetrically in the forebrain of the two turtles but not in that of the gecko.

## Materials and methods

### Recovery of reptile embryos

Fertilized eggs of the Chinese softshell turtle (*Pelodiscus sinensis*) and red-eared slider turtle (*Trachemys scripta elegans*) were obtained from Daiwa Farm (Taku, Saga, Japan) and Himeji City Aquarium, respectively. At the time of oviposition, turtle embryos were at the late gastrulation stage, which is slightly too early for the study of L-R patterning. The eggs were therefore incubated for 3–38 days (depending on the type of analysis) at room temperature (for early-stage embryos of Chinese softshell turtle), followed by setting at a temperature of 28° C (for late-stage embryos of Chinese softshell turtle), or at 28°C (red-eared slider turtle embryos) before recovery of embryos for analysis. Madagascar ground geckos (*Paroedura picta*) were maintained as described previously (Noro et al., 2009; Yoshida et al., 2016) by the Laboratory for Animal Resources and Genetic Engineering at the RIKEN Center for Biosystems Dynamics Research. At the time of oviposition, gecko embryos are at the 11- to 16-somite stage, when L-R asymmetric gene expression in the lateral plate has disappeared (Kajikawa et al., 2020). For examination of gene expression in the developing brain, gecko embryos were recovered from the oviduct before oviposition (9–10 days after the previous oviposition) or at 0–1 day postoviposition (dpo). Both turtle and gecko embryos were staged as described previously (Yntema, 1968; Hubert et al., 1985; Yoshida et al., 2016).

### *In situ* hybridization

Whole-mount *in situ* hybridization for turtle and gecko embryos was performed as described previously (Yoshida et al., 2016). The probes for *Nodal*, *Lefty*, and *Otx5* mRNAs were also performed as described previously (Yoshida et al., 2016). In the case of probes for *Pitx2*, *Not2*, and *Kctd12*, corresponding cDNA fragments were cloned from the embryos of turtle or gecko, and their nucleotide sequences were verified by Sanger sequencing. Sequences of the primers used for cloning and amino acid sequence homology of encoded genes are listed in Supplementary Table S1.

### Histological analysis

Nissl staining of the developing brain was performed with cresyl violet. To estimate the volume of the habenula, the area (mm^2^) of the whole habenula (or the lateral habenula and medial habenula individually) was measured with ImageJ for each Nissl section. The volume (mm^3^) of the habenula was estimated by multiplying the habenula area (mm^2^) in each section and the thickness of sections (10 μm). The area of the habenula region positive for *Kctd12* expression was similarly measured with ImageJ for each section and was expressed as an arbitrary unit.

### Immunofluorescence analysis

Turtle embryos were subjected to immunostaining with a monoclonal antibody to phosphorylated Smad2/3 (Cell Signaling Technology, catalog no. 3108) as described previously (Kawasumi et al., 2011; Kajikawa et al., 2020). Immunostained embryos were sectioned at a thickness of 8 µm and were observed with an FV1000 confocal microscope (Olympus).

### Molecular phylogenetic analysis

*Nodal*, *Lefty*, and *Pitx2* genes of reptiles were identified in the NCBI gene database (https://www.ncbi.nlm.nih.gov/gene). Their nucleotide sequences were obtained from the NCBI gene database or Reptiliomix (https://transcriptome.riken.jp/reptiliomix). The FoxH1-binding sequences (ASE-like sequences) in each gene were manually identified. Amino acid sequence homology of an encoded protein was analyzed by CLUSTALW (https://www.genome.jp//tools-bin/clustalw).

## Results

### L-R asymmetric expression of the *Nodal-Lefty-Pitx2* module in the developing forebrain of two turtle species

**FIGURE 1:**
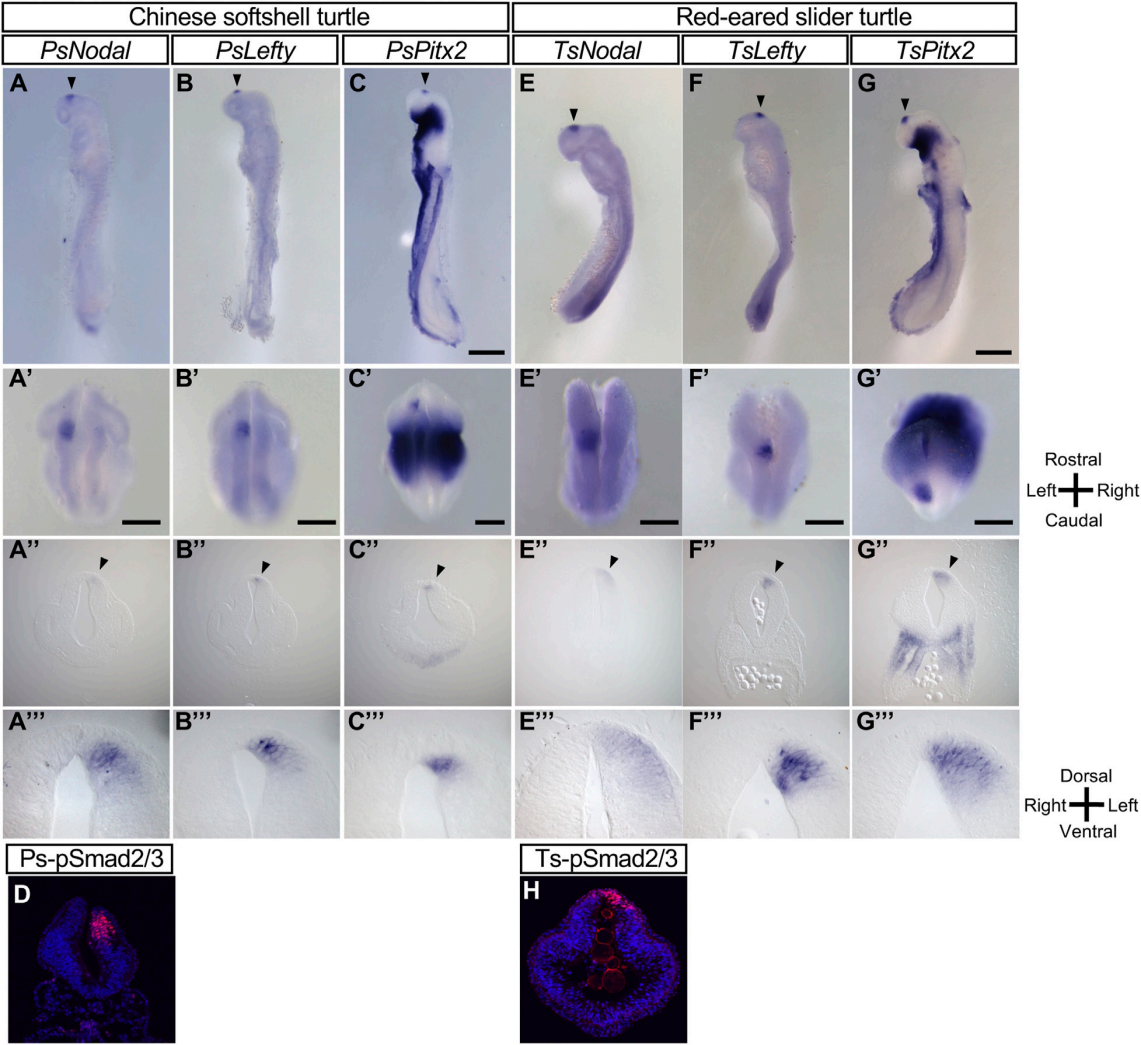
L-R asymmetric expression of *Nodal*, *Lefty*, *and Pitx2* in the developing brain of the Chinese softshell turtle and red-eared slider turtle. **(A–C)**, **(E–G)** Whole-mount *in situ* hybridization analysis of the expression domains of *Nodal* **(A,E)**, *Lefty* **(B,F)**, and *Pitx2* **(C,G)** in the developing brain of Chinese softshell turtle embryos at 3 dpo **(A–C)** and of red-eared slider turtle embryos at 4 dpo **(E–G)**. Top, middle, and bottom panels show left lateral views, dorsal views, and frontal sections, respectively. Domains manifesting L-R asymmetric expression are indicated by closed arrowheads. Rostral-caudal, dorsal-ventral, and L-R axes are indicated. Scale bars, 0.5 mm (top panels) and 0.2 mm (middle panels). Dorsal views, frontal sections, and higher magnification images of frontal sections of embryo in **(A–C)**, **(E–G)** are shown in **(A–C)**, **(E–G)**, **(A–C)**, **(E–G)**, and **(A–C)**, **(E–G)**, respectively. **(D,H)** Nodal activity in Chinese softshell turtle **(D)** and red-eared slider turtle **(H)** embryos at 3 and 4 dpo, respectively, as determined by immunofluorescence staining of phosphorylated (*p*) Smad2/3 (red fluorescence). Nuclei were stained with 4′,6-diamidino-2-phenylindole (blue fluorescence). *Nodal* activity is asymmetric (L > R) in the diencephalon.

When we previously examined *Nodal* expression in Chinese softshell turtle (*P. sinensis*) embryos (Kajikawa et al., 2020), we noticed that those at later developmental stages, after asymmetric expression of *Nodal* in the lateral plate mesoderm (LPM) had disappeared, showed left-sided *Nodal* expression in the forebrain (Figure 1A). Our present study revealed that *Lefty* (Figure 1B) and *Pitx2* (Figure 1C) are also expressed on the left side of the forebrain at similar stages. Frontal sections of these embryos confirmed left-sided expression of *Nodal*, *Lefty*, and *Pitx2* in the neural tube of the forebrain. The level of phosphorylated Smad2/3 has been shown to reflect the level of *Nodal* activity in mouse embryos (Kawasumi et al., 2011). Immunostaining with an antibody to phosphorylated Smad2/3 showed that *Nodal* activity is increased on the left side of the neural tube in *P. sinensis* (Figure 1D).

Given the marked asymmetric gene expression apparent in the forebrain of the Chinese softshell turtle, we examined another turtle species, the red-eared slider turtle (*T. scripta elegans*). *Nodal*, *Lefty*, and *Pitx2* all showed left-sided expression in the developing forebrain of this species (Figures 1E–G). *Nodal* signaling (as reflected by phosphorylated Smad2/3) was also activated on the left side of the forebrain (Figure 1H).

### Molecular and morphological L-R asymmetry in the turtle brain at later developmental stages

**FIGURE 2:**
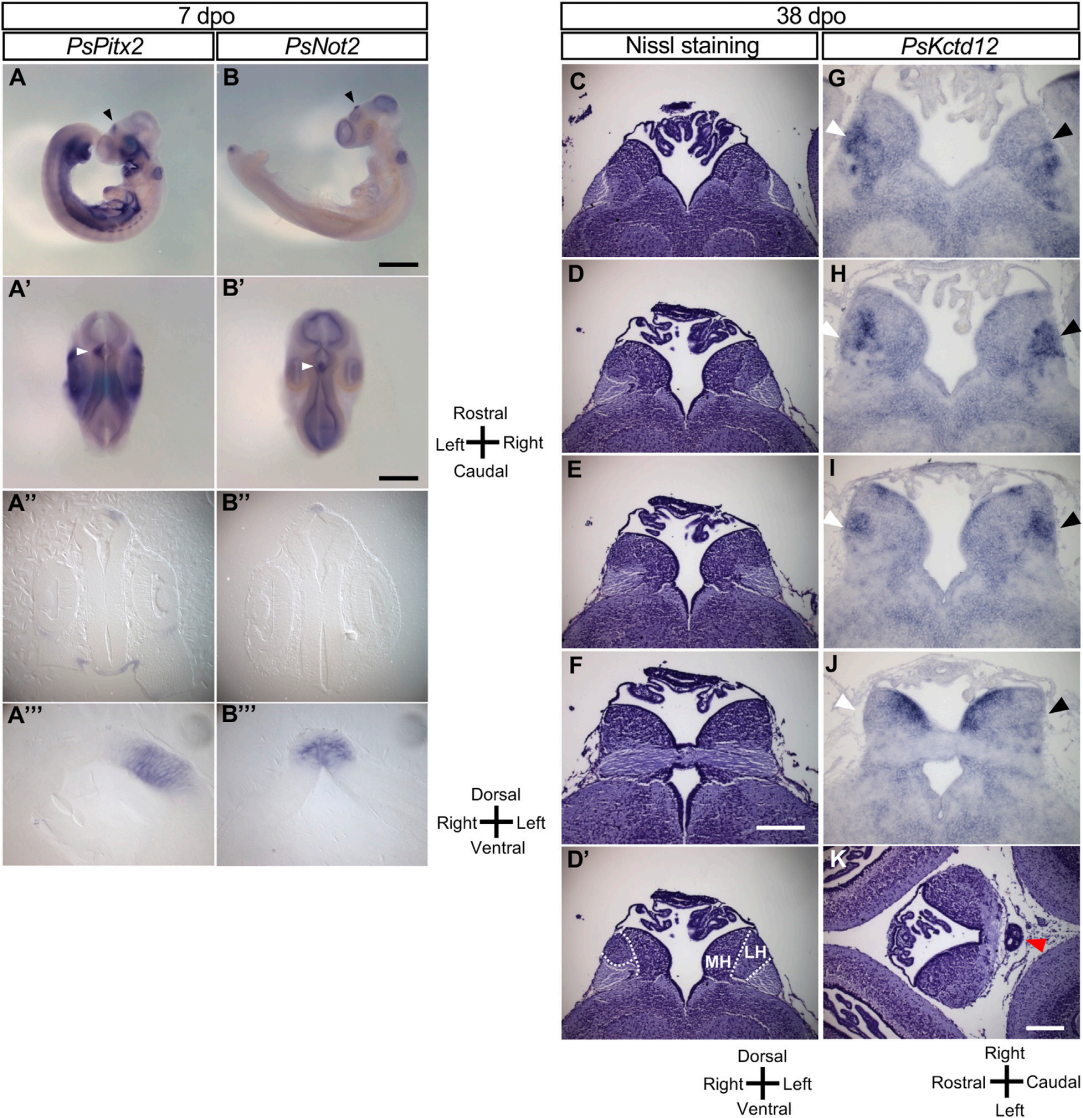
Molecular and morphological L-R asymmetry of the diencephalon in Chinese softshell turtle embryos at later stages. **(A,B)** Whole-mount *in situ* hybridization analysis of *Pitx2* **(A)** and *Not2* **(B)** in the developing brain of embryos at 7 dpo. Left lateral views (A,B), dorsal views **(A,B)**, frontal sections **(A,B)**, and higher magnification views of the frontal sections **(A,B)** are shown for each embryo. Closed arrowheads indicate the regions showing L-R asymmetric expression in the developing brain. Scale bars, 1.0 mm (top panels) and 0.5 mm (second from the top panels). **(C–F)** Nissl staining of frontal sections of embryos at 38 dpo. Sections at the level of the habenula are shown from the rostral side **(C)** to the caudal side **(F)**. There was no apparent L-R asymmetry in morphology although a subtle asymmetry may exist in **(D)**. Dotted lines in 2**(D)** indicate subdivision of the habenula shown in **(D)**. MH and LH denote the medial habenula and lateral habenula, respectively. Scale bar, 0.2 mm. **(G–J)** *Kctd12* expression in the habenula as examined by *in situ* hybridization with frontal sections of embryos at 38 dpo. Frontal sectional levels in **(G–J)** are similar to those in **(C–F)**, respectively. Regions positive for *Kctd12* expression on the right and left sides are indicated by white and black arrowheads, respectively. **(K)** Nissl-stained section of a Chinese softshell turtle at 38 dpo. The pineal organ is marked by the red arrowhead.

The L-R asymmetry of the pineal complex (composed of the pineal organ and parapineal organ) and the habenula varies among the vertebrate taxa. The pineal complex is derived from the dorsal region of the diencephalon. In zebrafish embryos, left-sided expression of *Nodal*, *Lefty*, and *Pitx2* occurs in the dorsal diencephalon and is followed by asymmetric expression of downstream genes such as *Nrp1* (neuropilin 1) and *Kctd12* (leftover) in the dorsal habenula (Gamse et al., 2003; Kuan et al., 2007). In the Chinese softshell turtle, asymmetric expression of *Pitx2* persists longer than that of *Nodal* and was apparent in the diencephalon even at 7 dpo (Figure 2A-2A‴). We obtained cDNA clones for *Not2* (a likely ortholog of *Flh* in zebrafish) and *Kctd12* from the Chinese softshell turtle, and examined the expression of these genes in the developing brain. In zebrafish, *Flh* expression marks the pineal organ at the midline and the parapineal located on the left side (Concha et al., 2003). In the Chinese softshell turtle, *Not2* was expressed at the dorsal side of the forebrain at 7 dpo (Figure 2B-2B‴), with this expression domain likely representing the future pineal organ in this species. In Nissl-stained sections of the Chinese softshell turtle at 38 dpo, the pineal organ was apparent, whereas the parapineal organ was not detected (Figure 2K). Unlike zebrafish, the habenula of the turtle consists of the lateral habenula and medial habenula on both sides (Figure 2D’), and there was no apparent L-R asymmetry in the morphology of the habenula (Figures 2C–F, Supplementary Figure S1). The volume of the habenula on the right and left sides was estimated from Nissl-stained sections (Supplementary Figure S1), which suggested no notable L-R difference (1.308 × 10^−2^mm^3^ for the left habenula vs 1.361 × 10^−2^mm^3^ for the right habenula). However, in some sections of the habenula (such as Figure 2D, 2D’), the lateral habenula on the left side looked larger than that on the right side.

In zebrafish, *Kctd12* is expressed in the dorsal habenula on the left side (Gamse et al., 2003; Kuan et al., 2007). In the Chinese softshell turtle, *Kctd12* expression was mainly found in the lateral habenula and showed a small but reproducible asymmetry at 38 dpo (Figures 2G–J; Supplementary Figure 2D–F). To quantify *Kctd12* expression, the area clearly positive for *Kctd12* expression in the habenula was measured for the left and right sides of a series of sections obtained from three turtle embryos. The total positive area was 116,883 (arbitrary units) for the left habenula and 95,188 for the right habenula in the embryo shown in Figure 2. In the remaining two embryos, the L-R ratio was 32,618 (left) vs 25,227 (right) or 31,603 (left) vs 28,425 (right), showing only a subtle difference. When sections of the habenula were carefully examined, however, there was a small but significant asymmetry depending on the level along the rostral–caudal axis. At the most rostral level, there was no obvious L-R difference in the location, shape, or size of positive areas (Figure 2G). At caudal levels (Figure 2H), the *Kctd12*-positive area was located in the lateral region of the lateral habenula on the left side, whereas it was found in the more medial region on the right side. At more caudal levels (Figure 2I), the location was symmetric but the expression level seemed higher on the left side. At the most caudal level (Figure 2J), bilateral *Kctd12* expression was only detected in the dorsal-medial habenula. A similar pattern of *Kctd12* expression was observed in the habenula of two other embryos (Supplementary Figure S2).

## Genes of the *Nodal-Lefty-Pitx2* module are not expressed asymmetrically in the developing forebrain of the Madagascar ground gecko

**FIGURE 3:**
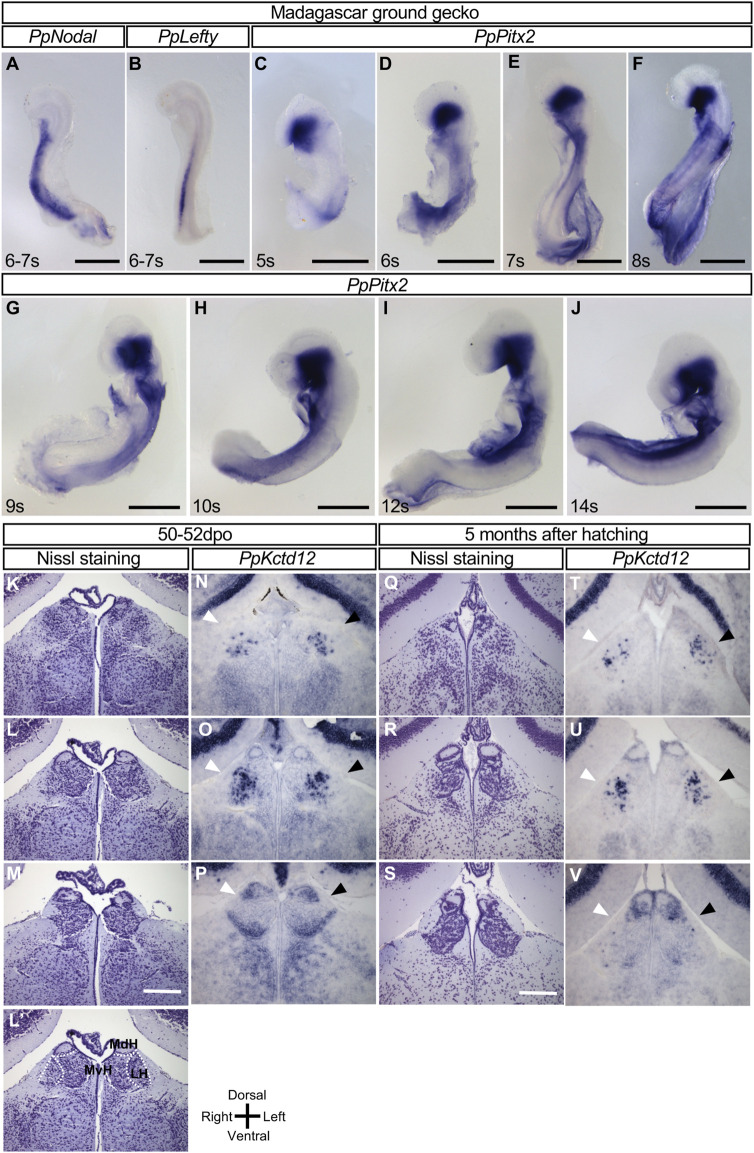
Lack of L-R asymmetric expression of *Nodal*, *Lefty*, and *Pitx2* in the developing brain of the Madagascar ground gecko. **(A–J)** Whole-mount *in situ* hybridization analysis of *Nodal* **(A)**, *Lefty* **(B)**, and *Pitx2* **(C–J)** expression in the developing brain of gecko embryos. These genes are not expressed in the developing brain, whereas their expression is apparent in other regions. The embryos were obtained before oviposition (9–10 days after the previous oviposition) **(A–F)** or at 0 to 1 dpo **(G–J)**. The number of somites (s) is indicated for each embryo. Scale bars, 0.5 mm. **(K–P)** The habenula of gecko at the 50–52 dpo. Nissl staining of frontal sections at the level of the habenula is shown from the rostral side **(K)** to the caudal side **(M)**. Dotted lines in (L′’) indicate the subdivision of the habenula shown in section (L). MdH, MvH, and LH denote the medial dorsal habenula, medial ventral habenula, and lateral habenula, respectively. *Kctd12* expression in the habenula as examined by *in situ* hybridization with frontal sections are shown in **(N–P)**. Frontal sectional levels in **(N)** to **(** *p* **)** are similar to those in **(K)** to **(M)**, respectively. Regions positive for *Kctd12* expression on the right and left sides are indicated by white and black arrowheads, respectively. **(Q–V)** The habenula of gecko at 5 months after hatching. Nissl staining of frontal sections at the level of the habenula are shown from the rostral side **(Q)** to the caudal side **(S)**. *Kctd12* expression in the habenula as examined by *in situ* hybridization with frontal sections are shown in **(T–V)**. Frontal sectional levels in **(T–V)** are similar to those in **(Q–S)**, respectively. Regions positive for *Kctd12* expression on the right and left sides are indicated by white and black arrowheads, respectively.

We next examined whether the L-R asymmetric expression of the *Nodal-Lefty-Pitx2* module in the developing brain is conserved among reptiles. With the use of cDNA clones for *Nodal*, *Lefty*, and *Pitx2* obtained from the Madagascar ground gecko (*P. picta*), we examined the expression of these genes in embryos of this species. In turtles, asymmetric gene expression in the developing brain was detected after that in the LPM had disappeared (Figure 1). Given that *Nodal* expression in the LPM of gecko embryos was detected before oviposition (Kajikawa et al., 2020), we recovered gecko embryos at various stages both before oviposition (9–10 days after the previous oviposition) and at 0 to 1 dpo. However, expression of neither *Nodal*, *Lefty*, nor *Pitx2* was detected in the forebrain at any stage examined (Figures 3A–J). Whereas asymmetric expression of a longer time, with this difference, is due to different enhancer sequences (Shiratori et al., 2001). However, *Pitx2* expression was not detected in the forebrain at any stage examined between the 5- and 14-somite stages (Figures 3C–J). These observations thus suggested that the *Nodal-Lefty-Pitx2* module is not asymmetrically expressed in the developing brain of the gecko.

We also examined the habenula of gecko embryos. The medial habenula was subdivided into dorsal-medial and ventral-medial habenula (Figure 3L’), but there was no L-R difference in the morphology or size at 50–52 dpo or 5 months after hatching (Figure 3K-M, 3Q-S, Supplementary Figure S3, S4). We have also examined *Kctd12* expression in the gecko habenula. *Kctd12* expression was detected in the habenula (mainly, the lateral habenula in the rostral region and the dorsal-medial habenula in the caudal region), but there was no notable asymmetry in its expression pattern at the two different stages (Figures 3N–P,T–V).

### The ASE of *Nodal*, *Lefty*, and *Pitx2* is conserved among reptiles

**FIGURE 4:**
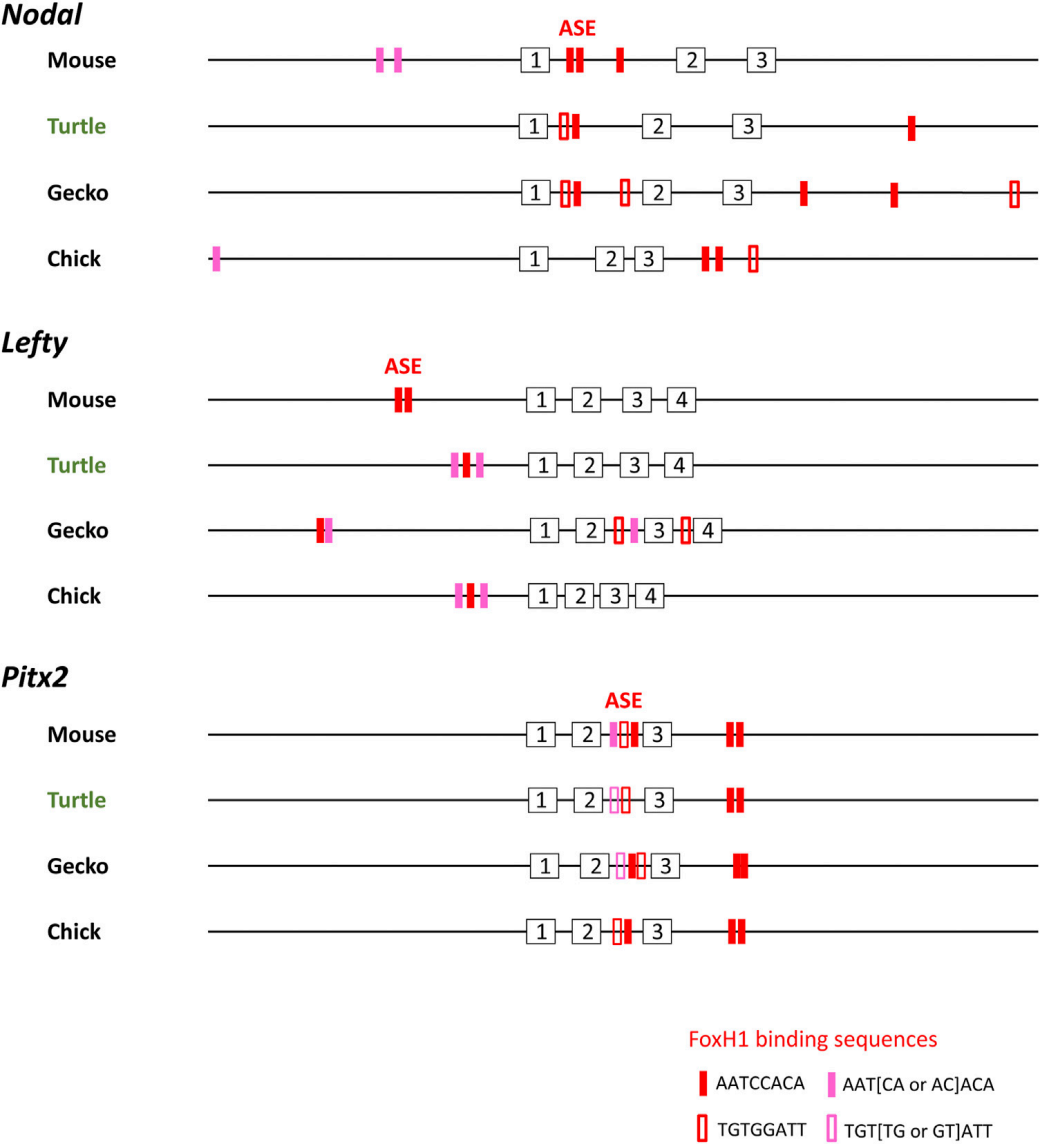
Transcriptional enhancer (ASE) sequences of *Nodal*, *Lefty*, and *Pitx2* genes of amniotes. FoxH1-binding sequences (red and pink boxes) present within a 30-kbp region of *Nodal*, *Lefty*, and *Pitx2* genes of mouse (*Mus musculus*), reptiles, and chicken (*Gallus*) are shown. Analysis of *Nodal* genes was previously performed (Kajikawa et al., 2020). For mouse, which has two *Lefty* genes, *Lefty2* is shown. Red closed and open boxes correspond to AATCCACA and TGTGGATT, respectively. Pink closed and open boxes denote ATT [AC or CA]ACA and TGT [GT or TG]ATT, respectively. Numbered boxes indicate exons. Turtle and gecko refer to the Chinese softshell turtle (*Pelodiscus sinensis*) and Madagascar ground gecko (*Paroedura picta*), respectively. *Nodal*, *Lefty*, and *Pitx2* genes of other turtles (green sea turtle and painted turtle) show similar configurations.

Asymmetric expression of *Nodal*, *Lefty*, and *Pitx2* in the LPM is highly conserved among vertebrates and is induced by Nodal signaling *via* a FoxH1-dependent enhancer (ASE). This, thus, appears to be the case for the LPM of fish, frog, and chick embryos. Simultaneous expression of *Nodal*, *Lefty*, and *Pitx2* in the developing forebrain of turtle embryos suggests that expression of these three genes is induced by the Nodal signaling. Indeed, a cluster of FoxH1-binding sequences resembling those of ASE is apparent in *Nodal*, *Lefty*, and *Pitx2* of the Chinese softshell turtle at conserved positions: in intron 1 for *Nodal*, in the upstream region for *Lefty*, and in intron 2 for *Pitx2* (Figure 4). ASE-like sequences are also present in the corresponding regions of *Nodal*, *Lefty*, and *Pitx2* of the Madagascar ground gecko (Figure 4).

## Discussion

The epithalamus shows L-R asymmetry of morphology, gene expression, and connectivity in many of the vertebrates studied, but its laterality varies among species. For instance, the habenula manifests varied L-R asymmetry in size, with the left side being larger or smaller than or equal in size to the right, depending on the species (Concha and Wilson, 2001). In the Chinese softshell turtle, the habenula showed no apparent asymmetry of size, whereas it did show a small but reproducible asymmetry in the subdivision pattern and *Kctd12* expression. The parapineal organ is also variable among vertebrate taxa, being present in some species but absent in others. Lizards possess a parietal eye, an organ equivalent to the parapineal organ of teleosts (Engbretson et al., 1981). In the Chinese softshell turtle, whereas the pineal organ was clearly evident, the parapineal was not detected. As previously suggested (Concha and Wilson, 2001), the connection between the parapineal organ and the left habenula does not necessarily correlate with asymmetries in size and organization of the habenula, and the parapineal organ may have been lost several times independently during evolution.

Our data suggest that asymmetric expression of the *Nodal-Lefty-Pitx2* module in the developing turtle forebrain is induced by *Nodal* signaling. If this is the case, then what might be the origin of the *Nodal* protein that initiates *Nodal* expression in the turtle brain? *Nodal* protein produced at the L-R organizer (blastopore) of turtle embryos may travel to the diencephalon, either by passive diffusion or by active transport, where it then activates the expression of the *Nodal* gene itself. A similar mechanism is thought to operate in mouse embryos, in which *Nodal* protein produced at the node is activated and then transported to the left side of the LPM, where it activates the *Nodal* expression (Oki et al., 2007). Alternatively, an unknown mechanism may initiate expression of *Nodal* on the left side of the turtle diencephalon, possibly similar to that by which *Nodal* expression is induced at the left side of the L-R organizer in chick and reptile embryos (Levin et al., 1995; Kajikawa et al., 2020). The distance between the L-R organizer and the forebrain is relatively large, however, and *Nodal* expression in the forebrain takes place about 1 day after that at the L-R organizer has disappeared. These spatial and temporal constraints between *Nodal* expression at the L-R organizer and that in the developing brain may leave the latter scenario also possible.

Why is the *Nodal-Lefty-Pitx2* module not expressed in the developing brain of other reptiles such as geckos and in that of mammals? If L-R asymmetric expression of *Nodal* in the brain, unlike that in the LPM, is indeed not induced by the *Nodal* derived from the L-R organizer, then the unknown signal that activates *Nodal* expression in the diencephalon of turtle embryos may be absent in other reptiles and in mammals.

The left-sided expression of *Nodal* in the LPM is highly conserved among vertebrates, without a single exception having been identified to date. In contrast, the expression pattern of the *Nodal-Lefty-Pitx2* module in the developing brain appears to be highly varied among vertebrates. Even within reptiles, turtles are positive, whereas the gecko is negative for the L-R asymmetric expression of this module. A phylogenetic tree of reptiles based on the genomic sequences of these genes and overall genomic sequences shows that the turtle is far distant from the gecko (Hara et al., 2018). In this regard, it will be of interest to see whether the alligator, a reptile positioned close to the turtle, also shows asymmetric gene expression in the developing brain.

## Data Availability

The raw data supporting the conclusion of this article will be made available by the authors on requests /Supplementary Material.

## References

[B1] Aizawa H. Amo R. Okamoto H. (2011). Phylogeny and ontogeny of the habenular structure. Front. Neurosci. 5, 138. 10.3389/fnins.2011.00138 22203792 PMC3244072

[B2] Bianco I. H. Wilson S. W. (2009). The habenular nuclei: A conserved asymmetric relay station in the vertebrate brain. Philos. Trans. R. Soc. Lond. B Biol. Sci. 364, 1005–1020. 10.1098/rstb.2008.0213 19064356 PMC2666075

[B3] Concha M. L. Burdine R. D. Russell C. Schier A. F. Wilson S. W. (2000). A nodal signaling pathway regulates the laterality of neuroanatomical asymmetries in the zebrafish forebrain. Neuron 28, 399–409. 10.1016/s0896-6273(00)00120-3 11144351

[B4] Concha M. L. Russell C. Regan J. C. Tawk M. Sidi S. Gilmour D. T. (2003). Local tissue interactions across the dorsal midline of the forebrain establish CNS laterality. Neuron 39, 423–438. 10.1016/s0896-6273(03)00437-9 12895418

[B5] Concha M. L. Wilson S. W. (2001). Asymmetry in the epithalamus of vertebrates. J. Anat. 199, 63–84. 10.1046/j.1469-7580.2001.19910063.x 11523830 PMC1594988

[B6] Engbretson G. A. Reiner A. Brecha N. (1981). Habenular asymmetry and the central connections of the parietal eye of the lizard. J. Comp. Neurol. 198, 155–165. 10.1002/cne.901980113 7229138

[B7] Gamse J. T. Thisse C. Thisse B. Halpern M. E. (2003). The parapineal mediates left-right asymmetry in the zebrafish diencephalon. Development 130, 1059–1068. 10.1242/dev.00270 12571098

[B8] Hara Y. Takeuchi M. Kageyama Y. Tatsumi K. Hibi M. Kiyonari H. (2018). Madagascar ground gecko genome analysis characterizes asymmetric fates of duplicated genes. BMC Biol. 16, 40. 10.1186/s12915-018-0509-4 29661185 PMC5901865

[B9] Hubert J. Seve A. P. Bouvier D. Masson C. Bouteille M. Monsigny M. (1985). *In situ* ultrastructural localization of sugar-binding sites in lizard granulosa cell nuclei. Biol. Cell 55, 15–20. 10.1111/j.1768-322x.1985.tb00404.x 2937491

[B10] Kajikawa E. Horo U. Ide T. Mizuno K. Minegishi K. Hara Y. (2020). Nodal paralogues underlie distinct mechanisms for visceral left-right asymmetry in reptiles and mammals. Nat. Ecol. Evol. 4, 261–269. 10.1038/s41559-019-1072-2 31907383

[B11] Kawasumi A. Nakamura T. Iwai N. Yashiro K. Saijoh Y. Belo J. A. (2011). Left-right asymmetry in the level of active Nodal protein produced in the node is translated into left-right asymmetry in the lateral plate of mouse embryos. Dev. Biol. 353, 321–330. 10.1016/j.ydbio.2011.03.009 21419113 PMC4134472

[B12] Kuan Y. S. Yu H. H. Moens C. B. Halpern M. E. (2007). Neuropilin asymmetry mediates a left-right difference in habenular connectivity. Development 134, 857–865. 10.1242/dev.02791 17251263

[B13] Lagadec R. Laguerre L. Menuet A. Amara A. Rocancourt C. Pericard P. (2015). The ancestral role of nodal signalling in breaking L/R symmetry in the vertebrate forebrain. Nat. Commun. 6, 6686. 10.1038/ncomms7686 25819227

[B14] Levin M. Johnson R. L. Stern C. D. Kuehn M. Tabin C. (1995). A molecular pathway determining left-right asymmetry in chick embryogenesis. Cell 82, 803–814. 10.1016/0092-8674(95)90477-8 7671308

[B15] Liang J. O. Etheridge A. Hantsoo L. Rubinstein A. L. Nowak S. J. Izpisua Belmonte J. C. (2000). Asymmetric nodal signaling in the zebrafish diencephalon positions the pineal organ. Development 127, 5101–5112. 10.1242/dev.127.23.5101 11060236

[B16] Noro M. Uejima A. Abe G. Manabe M. Tamura K. (2009). Normal developmental stages of the Madagascar ground gecko Paroedura pictus with special reference to limb morphogenesis. Dev. Dyn. 238, 100–109. 10.1002/dvdy.21828 19097047

[B17] Oki S. Hashimoto R. Okui Y. Shen M. M. Mekada E. Otani H. (2007). Sulfated glycosaminoglycans are necessary for Nodal signal transmission from the node to the left lateral plate in the mouse embryo. Development 134, 3893–3904. 10.1242/dev.009464 17913787

[B18] Roberson S. Halpern M. E. (2018). Development and connectivity of the habenular nuclei. Semin. Cell Dev. Biol. 78, 107–115. 10.1016/j.semcdb.2017.10.007 29107475 PMC5920772

[B19] Roussigne M. Bianco I. H. Wilson S. W. Blader P. (2009). Nodal signalling imposes left-right asymmetry upon neurogenesis in the habenular nuclei. Development 136, 1549–1557. 10.1242/dev.034793 19363156 PMC2675782

[B20] Shiratori H. Sakuma R. Watanabe M. Hashiguchi H. Mochida K. Sakai Y. (2001). Two-step regulation of left-right asymmetric expression of Pitx2: Initiation by nodal signaling and maintenance by Nkx2. Mol. Cell 7, 137–149. 10.1016/s1097-2765(01)00162-9 11172719

[B21] Signore I. A. Palma K. Concha M. L. (2016). Nodal signalling and asymmetry of the nervous system. Philos. Trans. R. Soc. Lond. B Biol. Sci. 371, 20150401. 10.1098/rstb.2015.0401 27821531 PMC5104501

[B22] Yntema C. L. (1968). A series of stages in the embryonic development of *Chelydra serpentina* . J. Morphol. 125, 219–251. 10.1002/jmor.1051250207 5681661

[B23] Yoshida M. Kajikawa E. Kurokawa D. Noro M. Iwai T. Yonemura S. (2016). Conserved and divergent expression patterns of markers of axial development in reptilian embryos: Chinese soft-shell turtle and Madagascar ground gecko. Dev. Biol. 415, 122–142. 10.1016/j.ydbio.2016.05.005 27174471

