# Supplementary material for: Left-right asymmetric expression of the Nodal-Lefty-Pitx2 module in developing turtle forebrain
Source: Front Cell Dev Biol. 2022 Oct 21;10:929808. doi: 10.3389/fcell.2022.929808 (PMC9634164; doi:10.3389/fcell.2022.929808)
Supplement: Supplementary file 1 [file DataSheet1.pdf]

Supplementary Materials

Supplementary Table 1. A list of primers used to clone reptile genes.

Sequences of the primers that were used to obtain cDNA clone for reptile genes are listed together with the size of an amplified sequence. Amino acid sequence homology of each gene, analyzed by CLUSTALW, is also indicated.

Gene	forward primers	reverse primers	amplicon (bp)	homolog score by CLUSTALW, amino acid sequence (%)		
Chinese softshell turtle				vs mouse	vs zebrafish	vs gecko
PsNodal	CTGGTCTCTCTCGTTCGACATGAGC	CAGTTGGCTTGAAGCTCTCATCCAC	748		36.2245 (bird-type)	49.2823
				30.226	29.4258 (mammalian-type)	
PsLefty	AACAGTGAAGTCACAATGGC	TCTCGGAAGTTGATGTAGTG	491	34.1598 (Lefty2)	59.6685 (Lefty2)	76.8595
PsPitx2	CAGCGACACTTCAGAGAGCA	AACACTGGCGTATCCAAAGC	787	94.3218	78.0255	77.918
PsNot2	GAGCTCCAGGCTGCCTGCTC	TGCCCGGCCAGCTATGATAC	467	25	39.7321	NA
PsOtx5	GGGTCTGAGCATGATGTCCT	CGCCTCACAAGACTTGGAAC	887	54.0107	78.0749	NA
PsKctd12	TCTTCCGCTACATCCTGGAC	CAGTCCCTGCAGAACACGTA	712	68.7117	63.0996 (Kctd12.2)	78.2209
					59.375 (Kctd12.1)	
Red-eared slider turtle				vs mouse	vs zebrafish	vs Chinese softshell turtle
TsNodal	TCCTGCTATCAGCCACTCCT	GGAGGACAACTTCCCCTTTC	716		38.2653 (bird-type)	74.6411
				31.0734	26.4019 (mammalian-type)	
TsLefty	GCCAGTGGATTAACCCAGAA	TGCTTTCCTCCTTGCAGTCT	730	33.6088 (Lefty2)	60.221 (Lefty2)	95.5923
TsPitx2	GTTGACGATCCCTCCAAGAA	GGGTCTGTCCACAGCGTATT	723	94.0063	78.9809	97.1609
Madagascar ground gecko				vs mouse	vs zebrafish	vs Red-eared slider turtle
PpNodal	GGAGGACCCCTCTGAGAACA	GTTGGCTGGAAGGTCTCATC	468		34.4388 (bird-type)	54.6729
				29.661	25.7403 (mammalian-type)	
PpLefty	GCTGARCTGAAGCTNTTYMARRARCC	GGYTCGATDAYCCARTAYTGNGTCC	503	31.7935 (Lefty2)	56.6298 (Lefty2)	77.4105
PpPitx2	GGCCACTATCACCACCAGAC	GCTTGGCTTTGAGTCTCAGG	811	76.3407	78.9809	76.6562
PpKctd12	TCCTCTTCCGCTACATCCTG	GCTGGTCCAGATCTTGTCGT	710	65.7492	62.3616 (Kctd12.2)	NA
					59.375 (Kctd12.1)	

Supplementary Figure 1

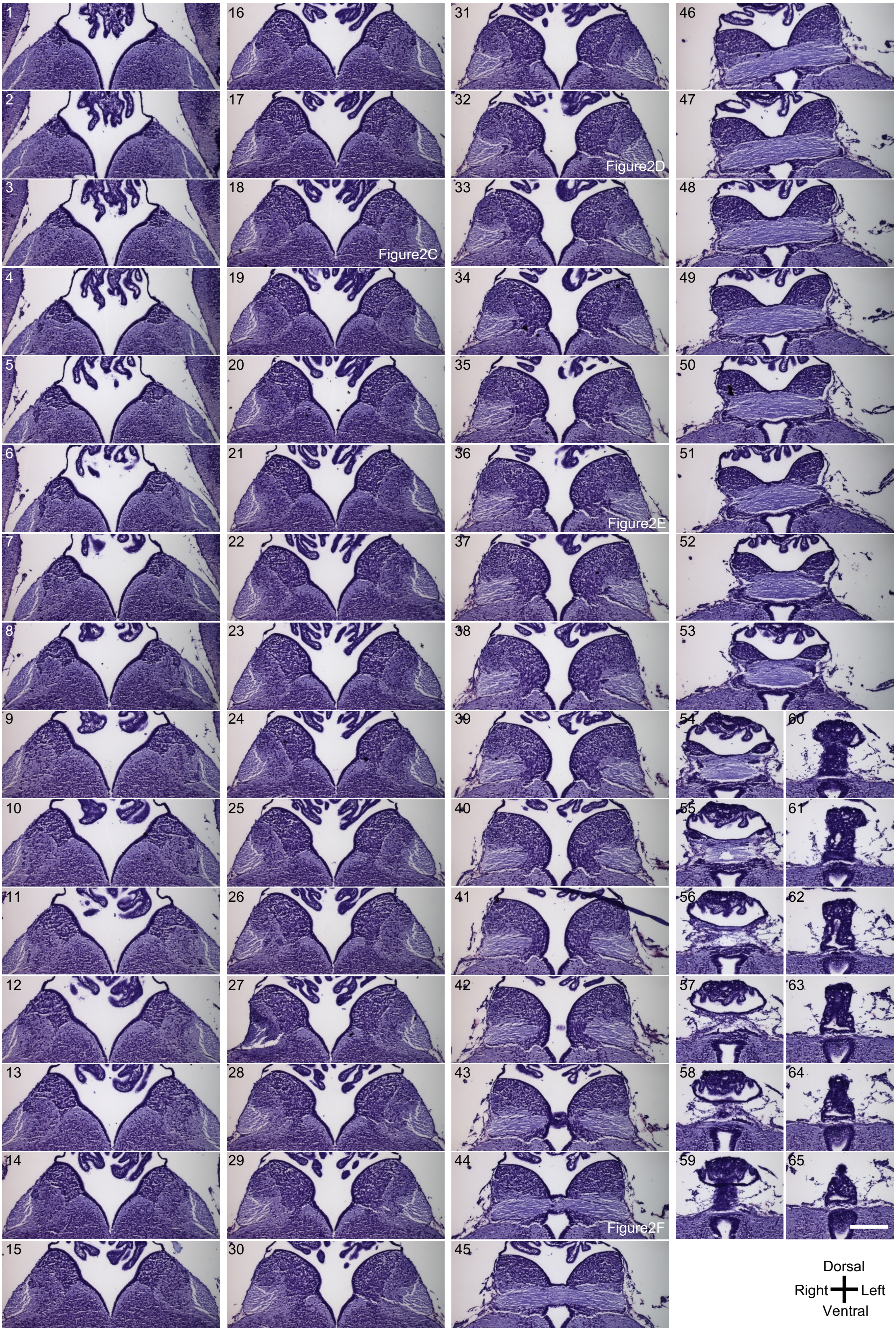

Supplementary Figure 1. A rostral-caudal series of Nissl stained sections of Chinese softshell turtle habenula at 38 dpo stage. Sections 1 and 65 correspond to the most rostral and caudal sections, respectively.

Supplementary Figure 2

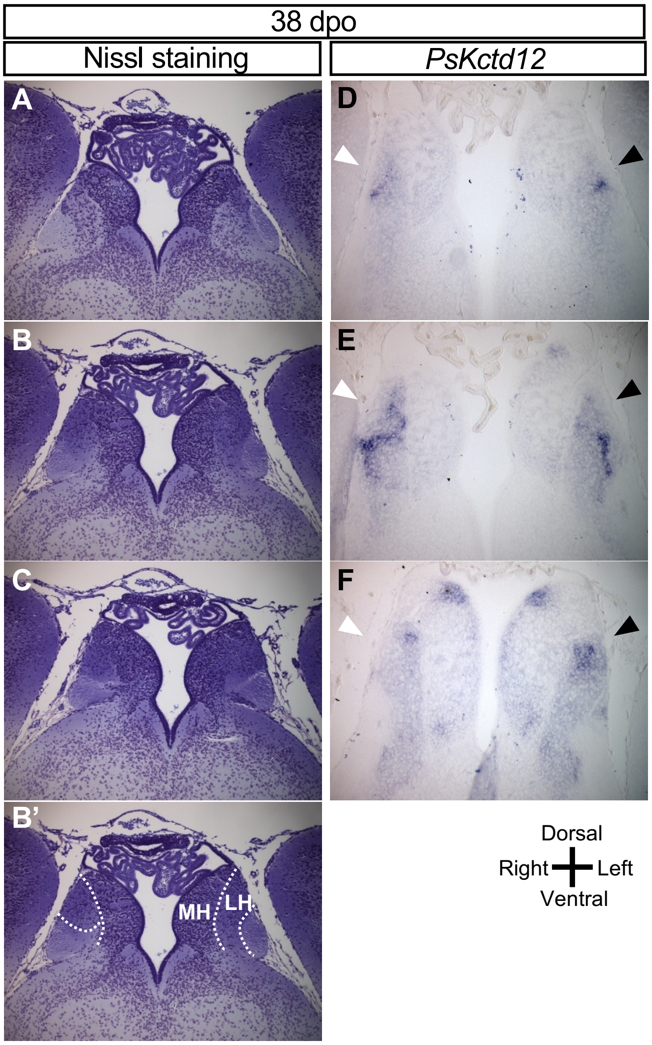

Supplementary Figure 2. Morphological and molecular L-R asymmetry of the habenula in Chinese softshell turtle at 38dpo. Nissl-stained sections (**A-C, B'**) of the habenula and *Kctd12* expression (**D-F**) are shown. Dotted lines in (**B'**) indicate subdividion of the habenula shown in (**B**). MH and LH denote the medial habenula and lateral habenula, respectively. Frontal sectional levels in **(D)** to **(F)** are similar to those in **(A)** to **(C)**, respectively, and are also similar to those in **(G)** to (**I**) of Figure 2, respectively. Regions positive for *Kctd12* expression on the right and left sides are indicated by white and black arrowheads, respectively.

Supplementary Figure 3

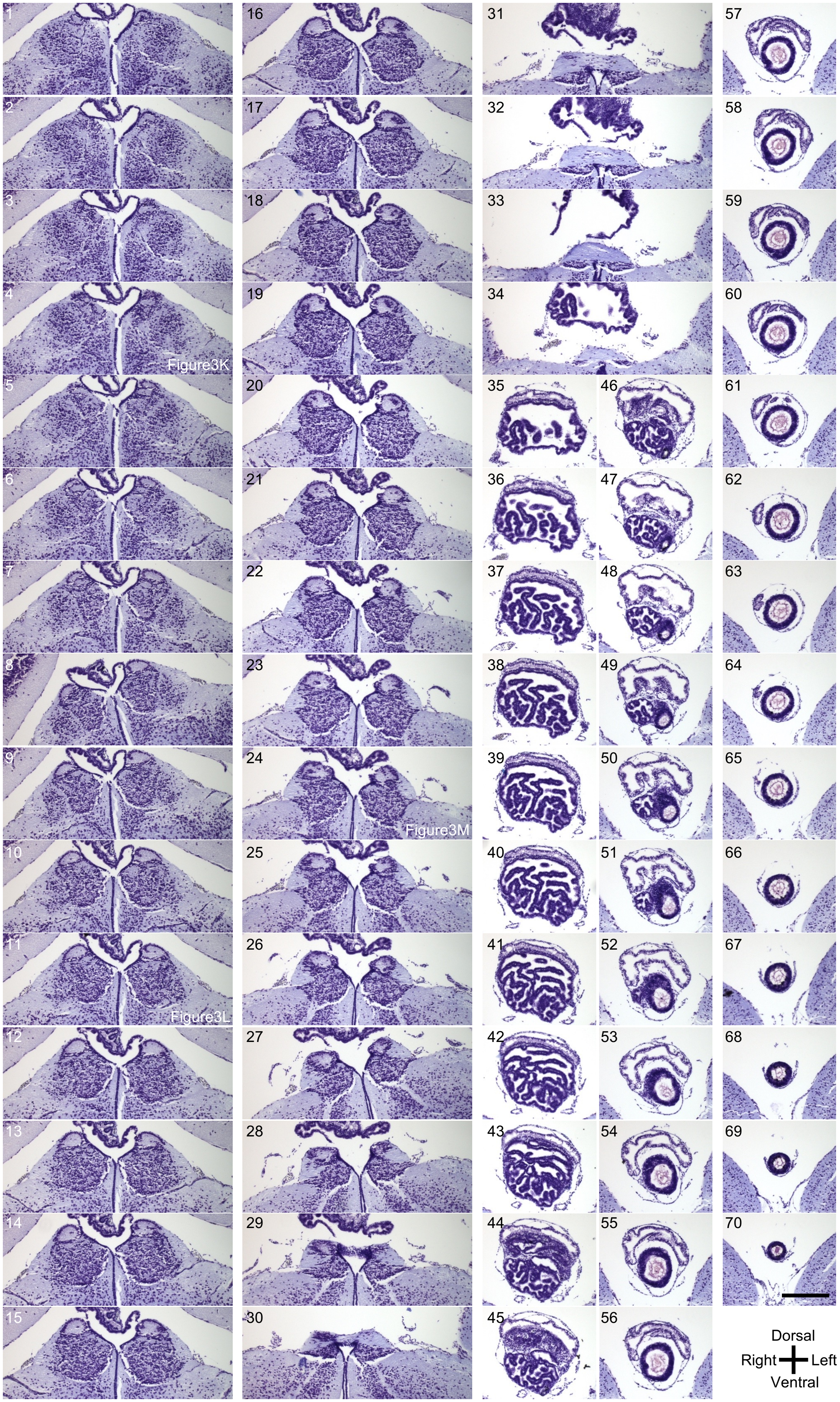

Supplementary Figure 3. A rostral-caudal series of Nissl stained sections of the gecko habenula at 50-52 dpo stage. Sections 1 and 70 correspond to the most rostral and caudal sections, respectively.

Supplementary Figure 4

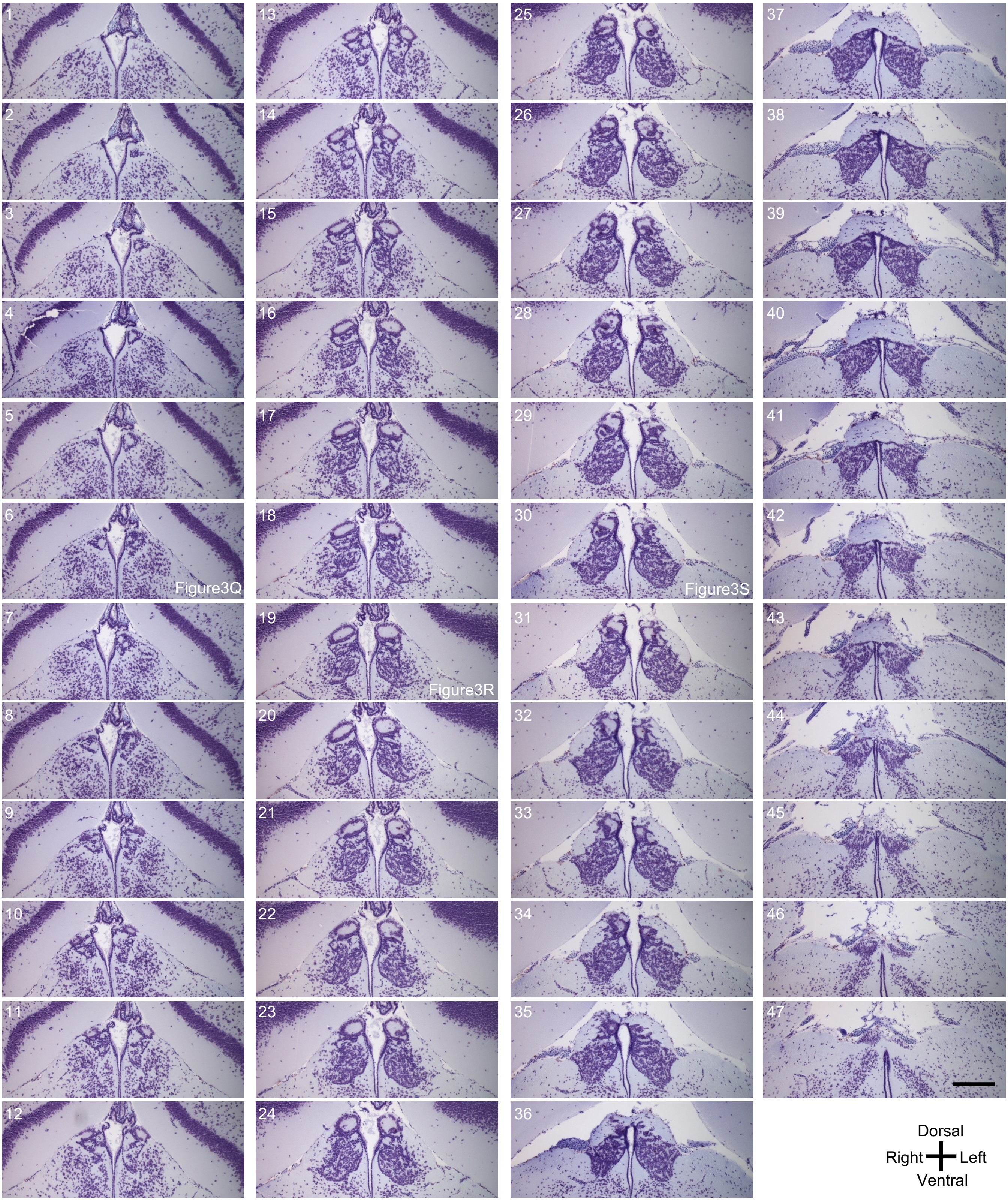

Supplementary Figure 4. A rostral-caudal series of Nissl stained sections of the habenula from a 5 month old gecko. Sections 1 and 47 correspond to the most rostral and caudal sections, respectively.
